# Chaetocin, a Natural Inhibitor of Transketolase, Suppresses the Non-Oxidative Pentose Phosphate Pathway and Inhibits the Growth of Drug-Resistant Non-Small Cell Lung Cancer

**DOI:** 10.3390/antiox14030330

**Published:** 2025-03-11

**Authors:** Song Li, Zhanying Lu, Wenli Jiang, Yao Xu, Ran Chen, Jie Wang, Binghua Jiao, Xiaoling Lu

**Affiliations:** 1 Department of Biochemistry and Molecular Biology, Naval Medical University, Shanghai 200433, China; lisonglisong0716@163.com (S.L.); jwlsally@163.com (W.J.); nmu-simba@foxmail.com (Y.X.); chenran199203@yeah.net (R.C.); 15159474237@163.com (J.W.); 2 Experimental Training Center of Basic Medical Science, Naval Medical University, Shanghai 200433, China; luzhanying2008@126.com

**Keywords:** chaetocin, drug target, transketolase, cisplatin resistance, non-small cell lung cancer

## Abstract

Worldwide, lung cancer is the most common cause of cancer-related death, which is made worse by the development of drug resistance during treatment. It is urgent to develop new therapeutic methods and small molecule drugs for tumor resistance. Chaetocin, extracted from *Chaetomium minutum*, is a natural compound with good antitumor activity. However, there are few studies on its tumor resistance. In this paper, firstly, chaetotocin significantly inhibited the viability and migration of cisplatin-resistant non-small cell lung cancer (NSCLC) cells and inhibited the xenograft growth of nude mice. Chaetocin at 4 mg/kg significantly inhibited A549/DDP xenograft growth with an inhibition rate of 70.43%. Subsequently, the underlying mechanism behind the actions of chaetocin was explored. It was discovered that chaetocin can inhibit transketolase (TKT), thereby inhibiting the growth of NSCLC cells and inducing cell death. Compared with cisplatin-sensitive cells, a lower concentration of chaetocin can inhibit cisplatin-resistance cell viability and migration. Mechanistically, TKT was identified as a potential target for chaetocin. The KD value of the interaction between chaetocin and TKT was 63.2 μM. An amount of 0.2 μM chaetocin may suppress the enzyme activity and expression level of TKT. We found the TKT expression is higher in cisplatin-resistant cells, which further explains why these cells were more vulnerable to chaetocin in terms of cell phenotype. Additionally, the muti-omics analysis and RNA interference suggested that chaetocin can inhibit the PI3K/Akt signaling pathway through TKT. In conclusion, chaetocin could directly bind to TKT, inhibiting its enzyme activity and expression, which interfered with intracellular metabolism and oxidation-reduction balance, and then regulated the PI3K/Akt signaling pathway to inhibit the growth of NSCLC and induce apoptosis.

## 1. Introduction

Lung cancer is one of the most common cancers and the primary culprit behind cancer-related fatalities across the world. Approximately 2.2 million new patients are diagnosed with lung cancer each year, and 75% of them die within five years of diagnosis [1]. With the continuous emergence of new technologies and new methods, targeted therapy and precision therapy of cancer have brought great hope to patients, but various new challenges have also emerged. Acquired drug resistance not only curtails the duration of the tumor’s response to treatment but also poses the most significant hurdle to achieving more substantial improvements in long-term survival within the realm of precision medicine [2].

Platinum antitumor compounds, such as cisplatin (DDP) and its analogs, have been used widely as a chemotherapeutic drug for a variety of malignancies including non-small cell lung cancer (NSCLC) [3]. During drug treatment, the tumor can rapidly and significantly fade in the initial stage of treatment, but the tumor often shows drug resistance in the later stage of treatment, which leads to treatment failure [4]. Metabolic reprogramming of tumor cells is one of the ten characteristics of tumors [5]. Metabolic reprogramming occurs not only in the process of transformation from normal cells to tumor cells but also in the development of advanced tumor cells, which is closely related to the sensitivity of anticancer drugs [6,7]. In recent years, many studies have found that the endogenous metabolic profile of tumor drug resistance also changes significantly, which is expected to reverse drug resistance by targeting metabolic changes [8,9,10].

Transketolase (TKT) played a critical role not only in directing carbon flux between nucleotide biosynthesis and glycolysis but also in modulating mitochondrial metabolism and function in adipocytes and immune cells [11]. In the human genome, three genes responsible for encoding TKT isoenzymes have been identified, namely, TKT, transketolase like 1 (TKTL1), and transketolase like 2 (TKTL2) [12]. TKT, the major type of transketolase, is expressed in normal human organs and most tumor tissues, while TKTL1 and TKTL2 are mainly expressed in the testis [13]. Many studies have shown that the activity of TKT in tumors may be related to tumor growth and metabolic regulation [14,15,16]. In addition, the expression of TKT is increased in tumor cells, and overexpression of TKT is associated with tumor invasion and poor prognosis [17]. TKT, as a potential therapeutic target, may provide new treatment strategies and methods by inhibiting the activity, affecting the growth of tumor cells and metabolic pathways.

**Figure 1:**
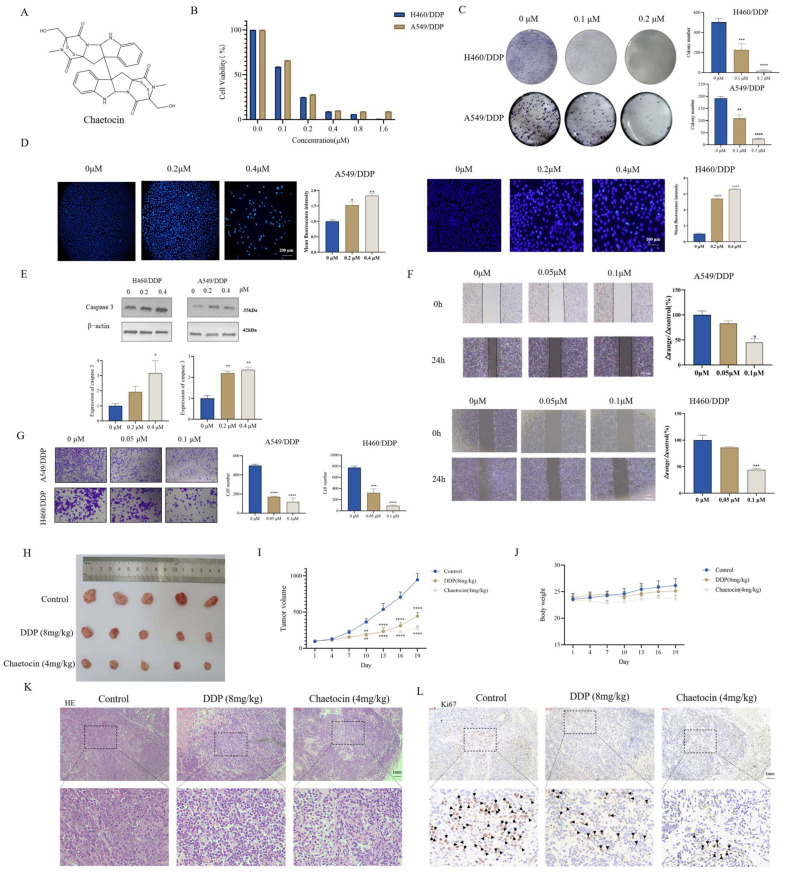
Chaetocin induced apoptosis in A549/DDP and H460/DDP and inhibited non-small cell lung cancer (NSCLC) growth. (**A**) Chemical structures of chaetocin. (**B**) Representative dose-response curve measured using the cell counting Kit-8 assay. A549/DDP and H460/DDP cell lines were exposed to chaetocin for 48 h. The concentration of chaetocin was taken as the *x*-axis; the *y*-axis represented the corresponding relative cell activity. (**C**,**D**) Representative images of colony-forming assays (**C**) and Hoechst staining experiments (**D**) after 48 h of treatment with chaetocin in A549/DDP and H460/DDP cells. (**E**) Western blotting analysis of caspase 3 in H460/DDP and A549/DDP cells. (**F**,**G**) Cell migration was determined via a wound healing assay (**F**) and transwell assay (**G**) upon chaetocin treatment for 24 h in A549/DDP and H460/DDP cells. (**H**) Chaetocin suppressed tumor growth in the A549/DDP xenograft mouse model. (**I**) The tumor volume of mice was measured using calipers (Shanghai, China) every three days as a marker for tumor growth. (**J**) The body weight of the mice was measured once every three days, serving as an indicator of tumor growth. (**K**,**L**) H and E (**K**) and IHC staining (**L**) results of tumors in the A549/DDP xenograft mouse model. The nucleus was bluish-purple. Cytoplasm and extracellular matrix were red. Ki67 was brown. * *p* < 0.05, ** *p* < 0.01, *** *p* < 0.001, **** *p* < 0.0001.

Natural products have been widely used in various cancer treatments for centuries. Chaetocin (Figure 1A) is an emerging secondary metabolite isolated from the fungi *Chaetomium minutum*, belonging to dimeric epi-3,6-dithio-2,5-diketopiperazines (ETPs) compounds. Like other ETP compounds, chaetocin has a unique transcyclic disulfide bond and possesses potent anti-inflammatory, immunosuppressive, and antitumor activities [18]. Chaetocin can inhibit the proliferation of various cancer cells by regulating multiple signaling pathways involved in tumorigenesis and progression, including endogenous and exogenous apoptosis, cell cycle arrest, migration and invasion inhibition, and cell proliferation [19]. However, chaetocin has been poorly studied in tumor-resistant cells, and its role remains unclear. In this paper, we found that a lower concentration of chaetocin can inhibit cisplatin-resistance cell viability compared with DDP-sensitive cells that have less expression of TKT. Chaetocin can directly bind to TKT, inhibiting its enzyme activity and expression, interfering with intracellular metabolism and oxidation-reduction balance, and then regulating the PI3K/Akt signaling pathway to inhibit the growth of NSCLC and induce apoptosis.

## 2. Materials and Methods

### 2.1. Reagents

DDP, chaetocin, and dimethylsulfoxide (DMSO) were obtained from Shanghai Yifei Biotechnology Co., Ltd. (Shanghai, China). Anti-transketolase, anti-ki67, anti-caspase 3, anti-Akt, and anti-PI3K antibodies were obtained from Cell Signaling Technology (Boston, MA, USA). Anti-β-actin and secondary antibodies were purchased from Wuhan Sanying Biotechnology Co., Ltd. (Wuhan, China). Matrigel (Thermo Fisher, St. Bend, OR, USA) was used. All other reagents were obtained from Sengon Biotechnology Co., Ltd. (Shanghai, China) unless stated otherwise.

### 2.2. Cell and Cell Culture

The human NSCLC cell lines A549 and cisplatin-resistant A549 cell line (A549/DDP) were purchased from Shanghai Fuheng Biotechnology Co., Ltd. (Shanghai, China). H460 and cisplatin-resistant H460 cell line (H460/DDP) were purchased from BIOWING Biotechnology Co., Ltd. (Shanghai, China). The human NSCLC cell line H520 was purchased from Shanghai Hongshun Biotechnology Co., Ltd. (Shanghai, China). In order to further cultivate cisplatin-resistant H520, this paper was referred [20]. Briefly, gradient cisplatin concentrations were used to treat H520. To maintain the cisplatin-resistant phenotype of H520, 1.5 μg/mL cisplatin was added to the medium. Cisplatin-sensitive cells were cultured with the RPMI-1640 medium supplemented with 10% fetal bovine serum (Gibco) and 1% Penicillin/Streptomycin (Gibco). A549/DDP or H460/DDP cells were cultured in a medium supplemented with 1 μg/mL DDP or 0.5 μg/mL, respectively, to maintain the resistant phenotype, and then further experiments were performed. Cells were incubated at 37 °C in a humidified environment with 5% CO_2_ in a humidified incubator.

### 2.3. Cell Viability

Cell viability was measured using the cell counting Kit-8 (Yuanye, Shanghai, China). After incubating for 12 h in 96-well plates, several concentrations of chaetocin were added to each well, and incubation was continued for another 48 h. Then, 10 μL of CCK-8 was added to each well, and cells were incubated for 1–4 h until the color of untreated controls turned orange. The absorbance was measured using a Gemini EM fluorometer at wavelengths of 450 nm.

### 2.4. Colony Formation

The colony formation assay was performed as reported previously [21]. Cells were plated at a density of 400 cells per well in 6-well plates and allowed to adhere overnight. Subsequently, the cells were exposed to varying concentrations of chaetocin for a period of 48 h. After this treatment, the culture medium was replaced, and the cells were further incubated for an additional 8 days until colonies became visible to the naked eye. To visualize the colonies, they were first fixed using 4% paraformaldehyde and then stained with crystal violet.

### 2.5. Wound Healing Assay

The motility of cells was measured using a wound healing assay as described previously [22]. Cells were seeded into 6-well plates and incubated for 12 h until they reached a confluence of approximately 90–100%. A 200 μL pipette tip was then used to create a scratch on the cell monolayer, simulating a wound. The wells were gently rinsed with PBS to remove any detached cells. Subsequently, various concentrations of chaetocin were added to the wells, and the cells were incubated with the drug for 48 h. At different time points (0 h and 24 h), images of the scratched area were captured using an inverted microscope at 4× magnification (Ningbo, China).

### 2.6. Transwell Migration Assay

Cells were plated in the upper chamber with serum-free neurobasal medium containing various concentrations of chaetocin and 500 μL of medium containing 20% FBS for 24 h of culture. Next, the cells in the transwell chamber were fixed with 4% paraformaldehyde for 15 min. After noninvaded cells in the upper chamber were removed by wiping, the remaining cells were stained with crystal violet staining solution for 10 min and washed with PBS. The cell migration was evaluated using microscopy.

### 2.7. Hoechst Staining

Hoechst 33342 staining was used to measure the cell death rate. The cells were incubated overnight in 6-well plates and then treated with chaetocin for 48 h. Subsequent treatments were performed according to the instructions. In brief, cells were washed with PBS and later fixed with the fixative solution for 10 min at room temperature. Finally, the Hoechst staining solution was incubated at room temperature for 15 min. The cells were observed and photographed with a fluorescence microscope. The photos were analyzed using ImageJ 1.54d software.

### 2.8. Quantitative Proteomics, RNA-Seq, and Bioinformatic Analysis

For RNA-Seq data, significant differentially expressed genes were identified based on the following criteria: |log2 fold change| > 1 and padj < 0.05. For quantitative proteomics, the *t*-test was used to compare the protein expression level between control and chaetocin-treated groups. A volcano plot was used to visualize significantly different proteins with a fold change greater than 1.2 or less than 0.8 and *p* < 0.05. Intersection analysis of down-regulated differential proteins and differential genes was performed using online tools Venny 2.1.0 “https://bioinfogp.cnb.csic.es/tools/venny/index.html (5 June 2024)”. Kyoto Encyclopedia of Genes and Genomes (KEGG) analysis was performed using online tools DAVID 2021 “https://david.ncifcrf.gov/ (5 June 2024)” with all Homo sapien genes as background.

### 2.9. Bioinformatics Analysis

UALCAN “https://ualcan.path.uab.edu/index.html (21 August 2023)” is an effective cancer data online analysis and mining site. It is mainly based on the analysis of related cancer data in the TCGA database, which can be used for related gene biomarker identification, expression profile analysis, survival analysis, etc. The protein and RNA expression levels of TKT in paracancerous tissues and lung cancer tissues were mined from the Clinical Proteomic Tumor Analysis Consortium (CPTAC) and TCGA plates of the UALCAN database, respectively. Using a Kaplan–Meier Plotter “https://kmplot.com/analysis/ (21 August 2023)”, the overall survival rate of high and low TKT in lung cancer was excavated.

### 2.10. Drug Affinity Responsive Target Stability (DARTS)

The procedure was performed as reported previously [23]. Cells were lysed and subsequently exposed to a range of chaetocin concentrations. After treatment, the cell lysates underwent digestion with pronase. Then, sodium dodecyl sulfate–polyacrylamide gel electrophoresis (SDS-PAGE) loading buffer was added to the digested samples. Following this, the prepared samples were separated via SDS–PAGE and visualized using silver staining. To identify the candidate proteins protected by chaetocin, Liquid Chromatography–Mass Spectrometry (LC–MS) was employed. The identity of these proteins was further verified through western blotting.

### 2.11. Cellular Thermal Shift Assay (CETSA)

Cells were exposed to DMSO or chaetocin for 48 h, collected, washed with PBS, aliquoted into PCR tubes, and heated in GeneExplorer at the indicated temperature for 3 min to denature proteins. The cells were then subjected to three freeze-thaw cycles with liquid nitrogen, and centrifuged at 20,000× *g* for 10 min at 4 °C. The supernatant was boiled in the loading buffer for western blotting.

### 2.12. Bio-Layer Interferometry (BLI)

The binding affinities of chaetocin and TKT were determined using a BLI assay via Octet K2 (Sartorius, Gottingen, Germany). Biotinized labeled TKT protein was loaded on the Streptavidin (SA) biosensors (ForteBio). For the background binding control, identical sensors are utilized and incubated in a protein-free buffer. Chaetocin is diluted in PBS. After washing the sensors and establishing a baseline with PBS supplemented with 2% DMSO, the biosensors are immersed in wells filled with varying concentrations of chaetocin to enable drug association. Subsequently, a dissociation step is carried out. All the assays are conducted in 96-well black microplates, with each well having a total volume of 200 μL. The experiments were performed at a temperature of 26 °C. All the data were analyzed using Octet data analysis 12.0 software.

### 2.13. Molecular Docking

Molecular docking was performed using Autodock vina and the crystal structure of human TKT was downloaded from the Protein Data Bank (PDB code 3MOS). During the docking process, proteins were regarded as rigid entities, while small molecules were deemed to be flexible.

### 2.14. Measurement of NADPH and ROS

The NADPH content of cells after chaetocin treatment was evaluated using an NADPH assay kit (Beyotime, Shanghai, China) according to the manufacturer’s instructions. Briefly, the cells were treated with extraction solution and centrifuged at 10,000× *g* at 4 °C for 5 min. Then the supernatant was incubated at 60 °C for 30 min to decompose the NADP+. After cooling on ice, the supernatant was reacted with the working solution at 37 °C in the dark for 20 min, and the absorbance was measured at 450 nm after a further 10 min of incubation with the addition of chromogenic agent.

Reactive oxygen species (ROS) were detected using the ROS detection kit. In short, the cells were seeded in 6-well plates. After 12 h, chaetocin was added for 12 h. The positive control reagent was added to the cells for 30 min before loading the probe. Cells were then incubated with diluted DCFH-DA (1:1000) for 1 h at 37 °C in the dark. A fluorescent microscope was used to take pictures of the cells. Image J software was used to analyze the photos and compare them to the control group.

### 2.15. Western Blotting

Total protein was extracted using a WholeCell Lysis Assay (KeyGEN Biotechnology, Nanjing, China). Protein concentration was determined with BCA reagent. The protein samples were separated using SDS–PAGE, transferred to PVDF membranes, and band blocked with 5% skimmed milk in Tris-buffered saline containing 0.05% Tween-20 at room temperature for 1 h. Then, the membranes were incubated with a transketolase antibody at 4 °C overnight and with horseradish peroxidase (HRP)-conjugated secondary antibodies for 1 h at room temperature. β-actin was used as the loading control. Finally, specific antibody binding was analyzed using Image Lab™ 4.0.1 Software on a ChemiDoc XRS+.

### 2.16. Lentivirus Infection to Establish Stable TKT-Knockdown Cell Lines

Lentivirus containing short hairpin RNA (shRNA) sequences against the TKT sequence (sh-TKT: CAGCATCTATAAGCTGGACAA) and control sequence (shNC: TTCTCCGAACGTGTCACGT) were purchased from GeneChem (Shanghai, China). The lentivirus was transfected into A549 and A549/DDP cells. An empty lentiviral vector served as the negative control. To obtain stable clones, cells were subjected to selection using 2 μg⋅ml^−1^ puromycin over a period of two weeks. Subsequently, the expression of TKT in the stably transfected clones was verified via western blotting.

### 2.17. Xenograft Studies

Animal studies were performed with the approval of the ethical committee of Naval Medical University and following relevant regulations and guidelines. Five-week-old male BALB/c nude mice were purchased from Shanghai Jihui Laboratory Animal Care Co., Ltd. (Shanghai, China). A total of 4 × 10^6^ A549/DDP cell suspensions were injected into the back subcutaneous of mice to induce transplanted tumors. The animals with tumor sizes between 80 and 100 mm^3^ were randomly divided into three groups with three individuals each. We measured the tumors once every three days using digital calipers, and calculated the volume of the tumor according to the following formula: (Width^2^ × Length) × 0.5. Mice were intraperitoneally injected with PBS, chaetocin 4 mg/kg, and DDP 8 mg/kg every three days. After 21 days of treatment, mice were humanely euthanized, and the tumor xenografts were dissected and measured. Heart, lung, kidney, and spleen tissue were collected, and the blood samples were collected after the mice’s eyeballs were removed. The removed tumors and tissues were fixed with 4% paraformaldehyde for subsequent Hematoxylin-eosin (HE) staining and Immunohistochemistry (IHC).

### 2.18. Statistical Analysis

Statistical analysis was performed using GraphPad Prism 9.0 software. The band intensities of western blotting were analyzed using Image J software. Data are presented as the mean ± SD. All experiments were independently repeated three times. For comparisons between two groups only, *t*-tests were employed. A two-tailed test was used to detect significance. In contrast, ANOVA was utilized when comparing multiple groups. Comparison between control and treatment groups was performed using the Dunnett test. Results with a *p* < 0.05 were considered statistically significant.

## 3. Results

### 3.1. Chaetocin Induced Apoptosis in A549/DDP and H460/DDP and Inhibited NSCLC Cell Growth

The sensitivity of cells to DDP was first determined using an CCK-8 assay (Supplementary Figure S1A–C). Chaetocin has a certain killing effect on normal cells (Supplementary Figure S1D). The cytotoxicity of chaetocin treatment with 24 h was not as good as that of 48 h (Figure 1B and Supplementary Figure S1E). To assess the cytotoxic effects, A549/DDP and H460/DDP cells were exposed to varying concentrations of chaetocin. The results revealed that chaetocin exhibited dose-dependent inhibitory effects on these cells. Specifically, at the 48-h time point, the IC_50_ of chaetocin for A549/DDP and H460/DDP cells was determined to be 0.13 ± 0.06 μM and 0.12 ± 0.02 μM, respectively (Figure 1B). The cell colony formation experimental results were consistent with CCK-8, chaetocin inhibited cell cloning formation in a concentration-dependent manner, and 0.1 μM chaetocin inhibited cell clone number formation (Figure 1C). Hoechst staining showed that 0.2 μM chaetocin could significantly induce apoptosis (Figure 1D). At the same time, we also found that the expression of caspase 3 increased with the increase of chaetocin concentration (Figure 1E). A wound healing assay and transwell assay were used to determine the effect of chaetocin on cell migration whose results showed that 0.1 μM chaetocin significantly inhibited cell migration in a serum-free medium for 24 h (Figure 1F,G). Next, the effects of chaetocin on the growth and formation of subcutaneous xenograft nodes derived from the A549/DPP in vivo in nude mice were investigated. Results showed that the tumor volume in the 4 mg/kg chaetocin group was significantly reduced compared with the control group, and the tumor weight in the chaetocin group was lower than that in the control group (Figure 1H,I and Supplementary Figure S1F). Compared with the control group, the mean tumor growth inhibition rates were 70.43 ± 6.25% and 56.40 ± 9.64%, respectively, in the 4 mg/kg chaetocin and 8 mg/kg DDP treatment groups (Supplementary Figure S1E). Histological analysis of tumor sections showed that the tumor cells in the chaetocin treatment group had significantly reduced nuclear polymorphism, swollen cells, loose cytoplasm, and light staining (Figure 1K). Immunohistochemical staining results indicated that the expression level of Ki67 in both the group treated with 4 mg/kg chaetocin and the group treated with 8 mg/kg DDP was notably lower compared to that in the control group (Figure 1L). There was no significant change in body weight (Figure 1J). Pathological sections showed that the structure of all organs was complete without obvious abnormalities, which suggested that chaetocin was safe and no fatal toxic side effects occurred (Supplementary Figure S1F). These results fully demonstrated that chaetocin can effectively inhibit the growth of NSCLC.

### 3.2. Chaetocin Significantly Inhibited PI3K/Akt Signaling Pathway

**Figure 2:**
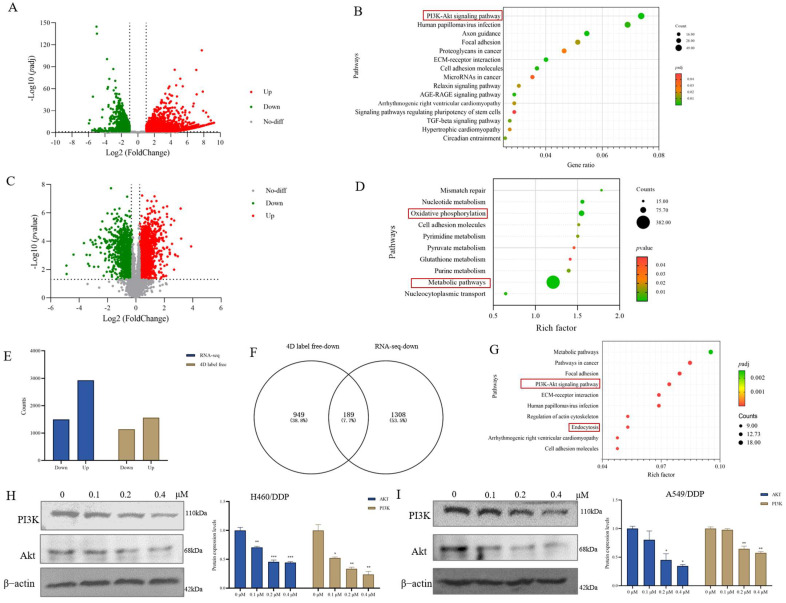
Chaetocin significantly inhibited the PI3K/Akt signaling pathway. (**A**,**C**) The volcano plots show differentially expressed genes (**A**) and proteins (**C**). Proteins that were significantly downregulated are represented by green circles, while those that were significantly upregulated are depicted by red circles in the plot. After A549/DDP cells were treated with chaetocin (0.2 μM, 48 h), the screening criteria for differential genes in the transcriptome were |log2 fold change| > 1 and padj < 0.05. The screening criteria for differential proteins in the proteome are fold change greater than 1.2 or less than 0.8 and *p* < 0.05. (**B**) Kyoto Encyclopedia of Genes and Genomes (KEGG) analysis of differential genes in the transcriptome of groups treatment with chaetocin or without chaetocin. The first 15 paths are shown. (**D**) KEGG analysis of different proteins in the proteome of groups treatment with chaetocin or without chaetocin. The top 10 paths are shown. (**E**) Bar chart based on up-regulated and down-regulated genes in the transcriptome and up-regulated and down-regulated proteins in the proteome. Blue represents the transcriptome, brown represents the proteome. (**F**) Venn diagrams are based on down-regulated genes from the transcriptome and down-regulated proteins from the proteome. (**G**) The KEGG bubble diagram of down-regulated genes from the transcriptome and down-regulated proteins from the proteome. (**H**,**I**) PI3K and Akt expression levels in H460/DDP and A549/DDP cells treated with different concentrations of chaetocin. * *p* < 0.05, ** *p* < 0.01, *** *p* < 0.001.

To elucidate the molecular mechanism of chaetocin, we analyzed the transcriptomic and proteomic changes of A549/DDP cells treated with chaetocin by RNA-seq and 4D label-free techniques. In the transcriptome, the similarity between untreated and treated groups was low (Supplementary Figure S2A). There were 5923 differential genes (|log2 fold change| > 1 and padj < 0.05), including 4426 up-regulated genes and 1497 down-regulated genes (Figure 2A). KEGG analysis showed that the differential genes were mainly concentrated in the PI3K/Akt signaling pathway. The score of ECM-receptor interaction also ranked high, which is consistent with cell migration in cell phenotype (Figure 2B). In the proteome, intra-group similarity was high, while inter-group similarity was low (Supplementary Figure S2B). A total of 2700 differential proteins were detected (fold change greater than 1.2 or less than 0.8 and *p* < 0.05), including 1562 up-regulated proteins and 1138 down-regulated proteins (Figure 2C). KEGG enrichment mainly focuses on oxidative phosphorylation and metabolic pathways (Figure 2D).

The transcriptomic down-regulated genes and proteomic down-regulated proteins were used to construct gene sets, and 189 genes were found to be consistent between the transcriptome and proteome (Figure 2E,F). These 189 genes were functionally annotated and enriched using KOBAS “http://bioinfo.org/kobas (11 June 2024)”. It was found that these genes were mainly concentrated on the metabolic pathway and the PI3K/Akt signaling pathway (Figure 2G). It is noteworthy that signaling pathways related to cell migration, such as ECM-receptor interaction, focal adhesion, and cell adhesion molecules, were also enriched to some extent. Western blot experiments also demonstrated that chaetocin significantly inhibited the PI3K/Akt signaling pathway in A549/DDP and H460/DDP cells in a concentration-dependent manner (Figure 2H,I). These results suggested that chaetocin could induce apoptosis by inhibiting the PI3K/Akt pathway.

### 3.3. TKT Was a Potential Target of Chaetocin in A549/DDP Cells

**Figure 3:**
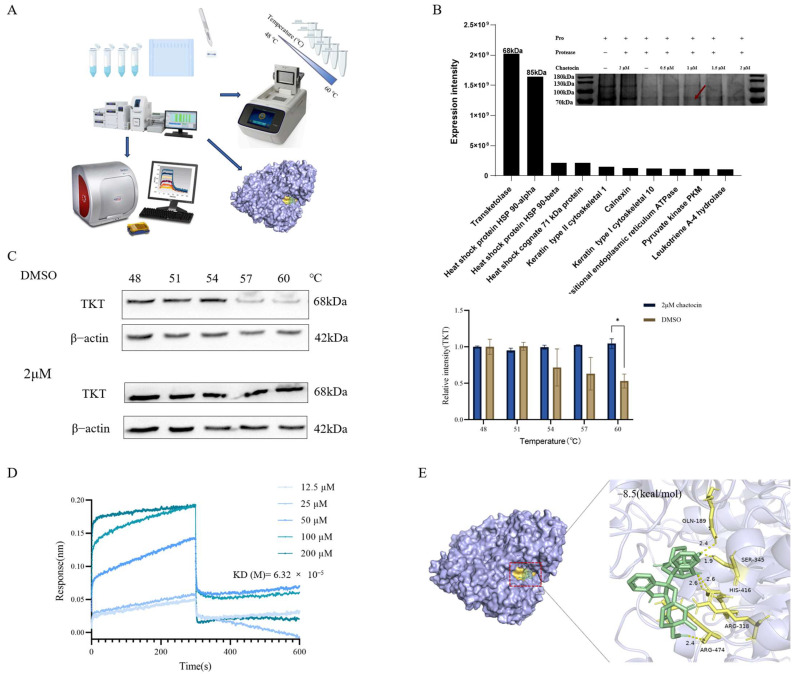
Transketolase (TKT) is a potential target of chaetocin in A549/DDP cells. (**A**) Flowchart of exploring the chaetocin target in A549/DDP cells. (**B**) Chaetocin protected TKT from proteolysis using a drug affinity responsive target stability assay (DARTS) analysis. The samples were separated via sodium dodecyl sulfate–polyacrylamide gel electrophoresis and visualized via Coomassie bright blue staining. A sample was taken where the arrow pointed to use for subsequent identification using mass spectrometry. (**C**) The supernatant of A549/DDP cell lysate, which had been incubated with either 2 μM chaetocin or dimethyl sulfoxide, was subjected to heating and centrifugation. Western blotting was performed on this supernatant. (**D**) Bio-Layer Interferometry analysis of chaetocin and TKT immobilized on a chip. (**E**) Predicted the combination pattern of chaetocin and human TKT (PDB code 3MOS). The surface of the protein was lavender carbon and hydrogen, and the ligands are shown in green as rods with carbon. The dashed yellow lines are hydrogen bonds. * *p* < 0.05.

Finding new drug targets has always been the focus and difficulty of global innovative drug research and development. As the source of innovative drug research and development, drug targets are of great significance to the development of innovative drugs. Therefore, we carried out the target research on chaetocin (Figure 3A). DARTS is a technique that can quickly and directly identify the potential target proteins of small molecule drugs. After the target protein is combined with the drug, the target protein is protected from protease hydrolysis, and then the activity and function of the target protein are maintained. First, DARTS was screened for proteins that directly bind to chaetocin. Coomassie blue staining revealed a prominent band at approximately 70 kDa in the cell lysates of those treated with chaetocin (Figure 3B). Four unique peptides of TKT were identified using LC–MS with the highest confidence and high enrichment, indicating that TKT was protected and enriched by chaetocin during protein hydrolysis (Figure 3B). TKT, a pivotal enzyme in the non-oxidative phase of the pentose phosphate pathway (PPP), plays a crucial dual role. It not only orchestrates the carbon flux between nucleotide biosynthesis and glycolysis but also modulates mitochondrial metabolism and function in adipocytes and immune cells [11]. CETSA results showed that the thermal stability of TKT was improved in chaetocin-treated cells compared with control cells, and the melting temperature curve was significantly different (Figure 3C). It suggested that chaetocin triggered TKT thermal stabilization through direct binding. DARTS and CETSA results were highly consistent. Subsequently, the KD value of the interaction between chaetocin and TKT was determined via BLI as 63.2 μM (Figure 3D). To further ascertain the binding site of chaetocin and TKT, Autodock vina was used for the molecular docking of TKT and chaetocin. The results showed that the hydroxyl and carbonyl on chaetocin formed hydrogen bonds with Gln-189, Ser-345, Arg-474, Arg-318, and His-416 on TKT, respectively (Figure 3E and Supplementary Figure S3). The binding affinity between TKT and chaetocin was predicted to be −8.5 kcal∙mol^−1^, reflecting a good binding potency. These results suggested that TKT was a potential target for chaetocin.

### 3.4. Chaetocin Decreased the Enzyme Activity and Expression Level of TKT

**Figure 4:**
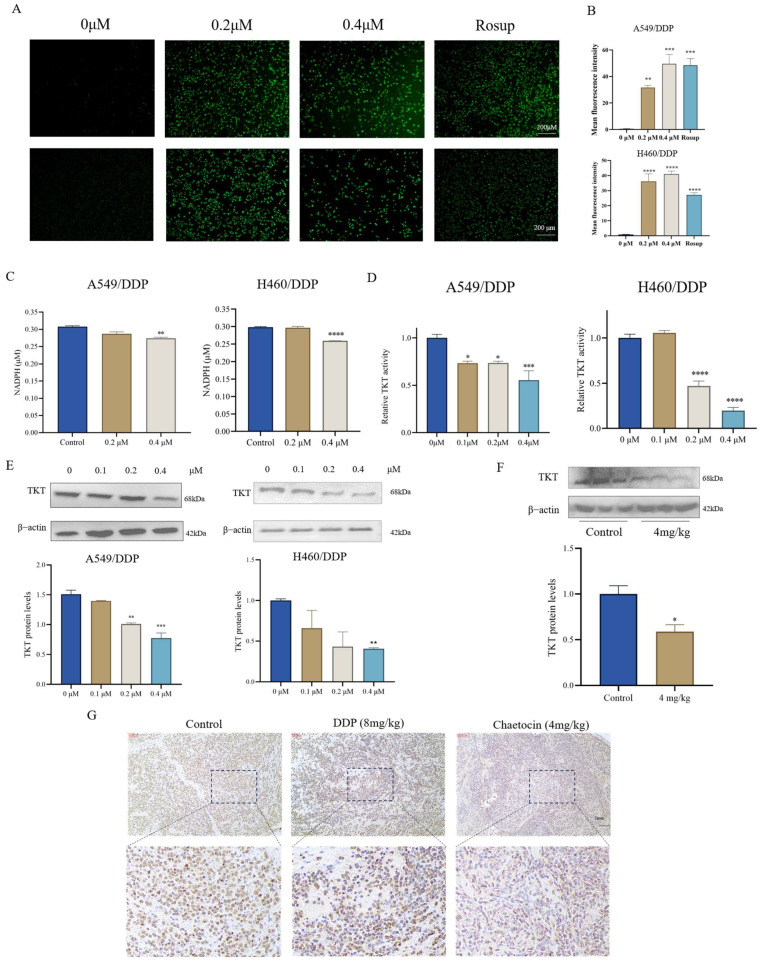
Chaetocin decreased the enzyme activity and expression level of TKT. (**A**) Representative fluorescent images from DCFH-DA probe showing ROS through prolonged exposure to chaetocin. (**B**) Quantitative evaluation of ROS fluorescence intensity. (**C**) NADPH production was assayed using the NADPH assay kit. (**D**) TKT enzyme activity was assayed via the NAD. (**E**) Western blotting analysis of TKT in A549/DDP and H460/DDP cells treated with chaetocin for 48 h. (**F**,**G**) WB (**F**) and IHC staining (**G**) results of tumors in the A549/DDP xenograft mouse model. TKT was brown. * *p* < 0.05, ** *p* < 0.01, *** *p* < 0.001, **** *p* < 0.0001.

The PPP is a fundamental component of cellular metabolism that is important for maintaining carbon homeostasis, supplying reducing molecules for anabolism, supplying precursors for the production of nucleotides and amino acids, and fending off oxidative stress. Higher ROS levels necessitate more NADPH to maintain a balance of reduction-oxidation states, in addition to the fact that DNA synthesis is active in tumor cells and demands a lot of reducing power [24]. Therefore, ROS and NADPH were used to indirectly show TKT enzymatic activity. After chaetocin treatment for 12 h, ROS was up-regulated during cell lysis (Figure 4A,B), indicating that the intracellular oxidation-reduction balance was broken. The level of NADPH decreased with the increase in chaetocin dose, indicating the PPP pathway disorder in tumor cells and decreased energy (Figure 4C). The results of TKT enzymatic activity showed that TKT tended to decrease significantly with the increase of chaetocin dose (Figure 4D). Western blotting showed that after treatment with chaetocin for 48 h, the TKT expression decreased with the dose increasing (Figure 4E). Immunohistochemical staining and WB analysis showed that the expression of TKT in the 4 mg/kg chaetocin group was significantly lower than that in the control group (Figure 4F,G). These results indicated that chaetocin decreased the activity and expression level of TKT.

### 3.5. Compared to Cisplatin-Resistant Cells, Cisplatin-Sensitive Cells Require Higher Chaetocin Concentrations to Inhibit

**Figure 5:**
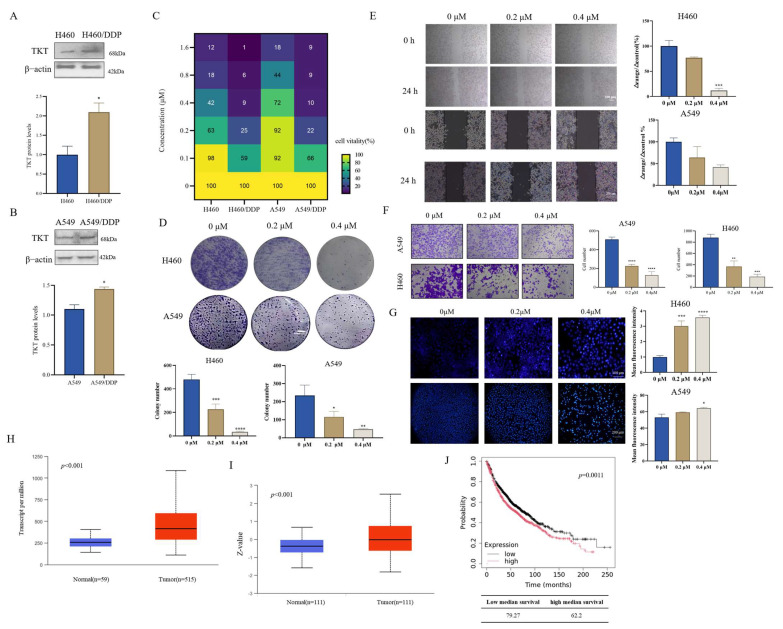
Compared to cisplatin-resistant cells, cisplatin-sensitive cells require higher chaetocin concentrations to inhibit. (**A**,**B**) Western blotting analysis of TKT expression level in H460/DDP (**A**) and A549/DDP (**B**) cells. (**C**) A representative dose-response curve was measured using the cell counting Kit-8 assay (Shanghai, China). H460, A549, A549/DDP, and H460/DDP cell lines were exposed to chaetocin for 48 h. The concentration of Chaetocin was taken as the *x*-axis; the *y*-axis represented the corresponding relative cell activity. (**D**) Representative images of colony-forming assays after 48 h of treatment with chaetocin in A549 and H460 cells. (**E**,**F**) Cell migration was determined using a wound healing assay (**E**) and transwell assay (**F**) upon chaetocin treatment for 24 h in A549 and H460 cells. (**G**) Hoechst staining assay detected the apoptosis of A549 and H460 cells treated with chaetocin for 48 h. (**H**) Expression of TKT in lung adenocarcinoma from TCGA samples. (**I**) Protein expression of TKT in lung adenocarcinoma from CPTAC samples. (**J**) Kaplan–Meier curve. * *p* < 0.05, ** *p* < 0.01, *** *p* < 0.001, **** *p* < 0.0001.

Owing to its distinct metabolic influence, the inhibition of TKT holds the potential to foster tumor cell apoptosis through the elevation of ROS levels. Concurrently, it can hinder tumor proliferation by diminishing DNA synthesis. These mechanisms underscore the crucial role that TKT inhibition plays in tumor therapy and combating chemotherapy resistance [24]. First, WB results showed that the expression of TKT in A549/DDP and H460/DDP was higher than that in A549 and H460 (Figure 5A,B), indicating that TKT was over-activated during the formation of tumor chemotherapy resistance. The IC_50_ of chaetocin to A549 and H460 was 0.68 ± 0.04 μM (IC_50(A549/DDP)_ = 0.13 ± 0.06 μM) and 0.33 ± 0.02 μM (IC_50(H460/DDP)_ = 0.12 ± 0.02 μM), respectively, which are about a five-fold and a three-fold difference from the IC_50_ of DDP-resistant cells, respectively (Figure 5C). The results of the cloning formation assay showed that chaetocin inhibited DDP-sensitive cell cloning formation in a concentration-dependent manner, and 0.2 μM chaetocin slightly inhibited cell clonal formation (Figure 5D), which is quite different from the obvious inhibition of colony formation of DDP-resistant cells by chaetocin at 0.1 μM. Wound healing assay, transwell assay, and Hoechst staining also showed that chaetocin could dose-dependently inhibit cell migration (Figure 5E,F) and induce apoptosis (Figure 5G). However, DDP-sensitive cells require a higher concentration of chaetocin to achieve the same effect as DDP-resistant cells. These cell phenotype experiments also fully demonstrated that cisplatin-sensitive cells require higher chaetocin concentrations to inhibit compared to cisplatin-resistant cells, which also indicated that the killing effect of chaetocin on cells was related to the content of TKT. Clinical data showed that the expression level of TKT in tumor tissues was higher than that in adjacent tissues (Figure 5H,I), and the expression level of TKT was negatively correlated with overall survival (Figure 5J). High levels of TKT are detrimental to the prognosis of patients. Chaetocin may be more effective in lung cancer patients with high TKT expression.

### 3.6. Knocking Down TKT Could Reduce the Effect of Chaetocin-Inducing Apoptosis

**Figure 6:**
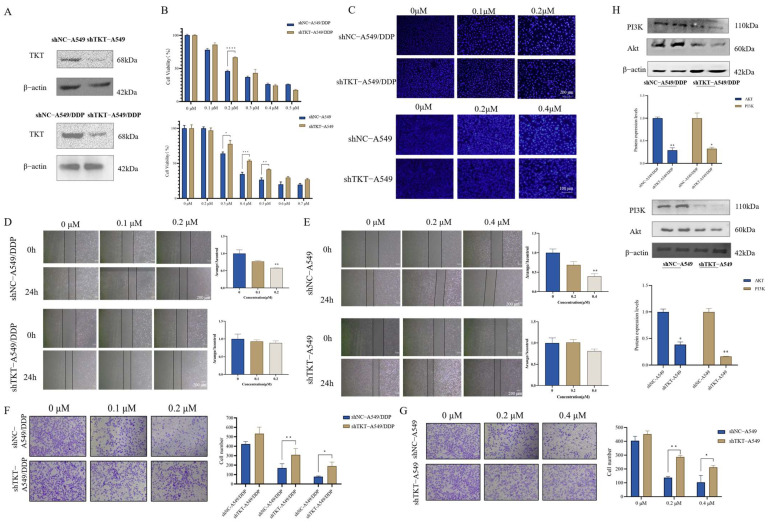
Knocking down TKT could reduce the effect of chaetocin-inducing apoptosis. (**A**) After puromycin selection, Western blotting was conducted to detect the TKT protein levels in A549 and A549/DDP cells that had been transfected with either shNC or shTKT lentivirus. (**B**) The viability of A549/DDP and A549 cells transfected with shNC or shTKT lentivirus was assessed following a 48 h exposure to chaetocin. (**C**) Representative images of Hoechst staining experiments after 48 h of treatment with chaetocin in A549/DDP and A549 cells transfected with shNC or shTKT lentivirus. (**D**,**E**) The wound healing assay for cell migration was performed in A549/DDP (**D**) and A549 (**E**) cell lines transfected with shNC or shTKT lentivirus with the treatment of chaetocin at the indicated concentrations for 24 h. (**F**,**G**) The transwell assay for cell migration was performed in A549/DDP (**F**) and A549 (**G**) cell lines transfected with shNC or shTKT lentivirus with the treatment of chaetocin at the indicated concentrations for 24 h. (**H**) Western blotting analysis of PI3K and Akt in A549/DDP and A549 cells transfected with shNC or shTKT lentivirus. * *p* < 0.05, ** *p* < 0.01, *** *p* < 0.001, **** *p* < 0.0001.

To further explore the effect of target TKT on chaetocin apoptosis induction, the effect of TKT knockout by transfecting lentivirus-containing shRNA into cancer cells was tested by constructing a stable TKT-knockout low-cell line. TKT was knocked down in both A549 and A549/DDP cells (Figure 6A). After 48 h treatment with chaetocin, the cell viability of the knockdown group was higher than that of the control group. IC_50_ of shTKT-A549, shNC-A549, shTKT-A549/DDP, and shNC-A549/DDP were 0.48 ± 0.13 μM, 0.38 ± 0.03 μM, 0.24 ± 0.04 μM and 0.19 ± 0.01 μM, respectively (Figure 6B). In addition, the effects of chaetocin on cell migration inhibition and apoptosis induction were also impaired after TKT knockdown (Figure 6C–G). We also found that TKT knockdown inhibited the PI3K/Akt signaling pathway in cells (Figure 6H). These data suggested that TKT knockdown weakened chaetocin-mediated NSCLC inhibition, further supporting TKT as a functional target for chaetocin inhibition of NSCLC.

## 4. Discussion

**Figure 7:**
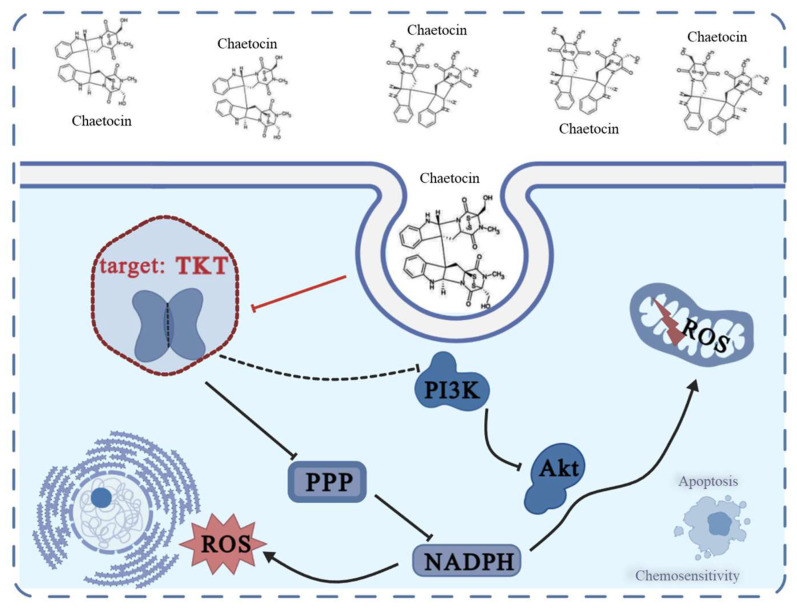
The mechanism overview of chaetocin.

Lung cancer is the leading cause of cancer morbidity and mortality in 2022 [25]. Currently, DDP and carboplatin are the platinum compounds used in patients with NSCLC. Drug resistance that develops during treatment is one of the reasons why lung cancer patients have low survival rates [23]; therefore, it is critical to develop new therapeutic approaches and new small-molecule drugs for tumor resistance. Chaetocin, with its unique transcyclic disulfide bond, has strong anti-inflammatory, immunosuppressive, and anti-tumor properties [18]. Numerous studies have demonstrated that chaetocin is also a sensitizer of certain apoptosis-inducing factors, which can cause glioblastoma cell apoptosis. To put it another way, chaetocin may be able to withstand tumor medication resistance [26]. Here we mainly focused on H460 and A549 and the corresponding cisplatin resistant cell lines because we detected the cytotoxicity of chaetocin against H520 and H520/DDP and found that the IC_50_ of chaetocin against H520 and H520/DDP is 0.25 ± 0.02 μM and 0.19 ± 0.02, respectively. The IC_50_ of chaetocin to A549 and H460 was 0.68 ± 0.04 μM (IC_50(A549/DDP)_ = 0.13 ± 0.06 μM), and 0.33 ± 0.02 μM (IC_50(H460/DDP)_ = 0.12 ± 0.02 μM), respectively, which are about a five-fold and a three-fold difference from the IC_50_ of DDP-resistant cells, respectively (Figure 5C)). We further detected the expression of TKT in H520 and H520/DDP cells and found that although the TKT content in H520/DDP cells increased, there was no significant difference between the TKT content in H520 cells and that in H520 cells. In this paper, chaetocin can bind to TKT, preventing its expression and enzyme activity, disrupting intracellular metabolism and oxidation-reduction balance, and subsequently controlling the PI3K/Akt signaling pathway to inhibit NSCLC growth and trigger apoptosis (Figure 7).

First of all, we discovered that DDP-resistant cells react to lower concentrations of chaetocin more strongly compared with DDP-sensitive cells. Multi-omics analysis revealed that chaetocin’s impact on the PI3K/Akt signaling pathway was the most significant. PI3K/AKT is an important pathway involved in tumorigenesis, proliferation, apoptosis, angiogenesis, epithelial-mesenchymal transformation, immune microenvironment, and therapeutic resistance [27]. The PI3K/Akt pathway is a desirable target for therapeutic approaches because of the association between its activation and cancer. Hayakawa and coworkers employed genetic techniques and small chemical inhibitors to confirm that DDP’s stimulation of Akt activation was the cause of the observed chemotherapeutic resistance [28]. When small cell lung cancer (SCLC) relapsed, genes involved in the PI3K/Akt signaling pathway were enriched for high frequency acquired copy number variations or acquired somatic mutations [29]. According to transcriptomic analysis of SCLC cell lines found in public databases, drug-resistant SCLC cell lines had an up-regulated PI3K/Akt pathway. Modulating the PI3K/Akt/mTOR signaling pathway might alter the effect of gefitinib in treating esophagus cancer [30]. Guajadial may be a viable strategy for overcoming drug resistance during chemotherapy of breast cancer, according to Li et al.’s findings that treatment with Guajadial suppressed the activation of the PI3K/Akt pathway in drug-resistant breast cancer cells [31]. Yu et al. suggested that the PI3K/Akt/NF-kB pathway could mediate epithelial-mesenchymal transition (EMT) by downregulating the expression of E-cadherin, leading to tumor drug resistance [32]. Therefore, the PI3K/Akt pathway is one of the possible targets for natural compounds to enhance the cytotoxicity of chemotherapy drugs.

Furthermore, we conducted drug target research because drug targets can provide direction and opportunities for the development of innovative drugs. The results suggested that TKT was a potential target of chaetocin in NSCLC. Simultaneously, we would like to highlight an important observation from our DARTS data, which indicates that Heat Shock Protein 90 (HSP90) ranks second in terms of enrichment degree, surpassed only by TKT. HSP90, a crucial molecular chaperone involved in the folding, stabilization, and functional regulation of numerous cellular proteins, plays a pivotal role in various oncogenic processes. Notably, prior studies have demonstrated that chaetocin binds to the C-terminal domain of HSP90α in leukemia, disrupting the function of several HSP90 client proteins and subsequently activating the proteasome pathway to degrade SUV39H1 [33]. Furthermore, HSP90 has been implicated in chemotherapy resistance in osteosarcoma cells, where it inhibits apoptosis via the JNK/P38 pathway and promotes autophagy through the PI3K/Akt/mTOR pathway [34]. Given these findings, we are particularly intrigued by the potential interplay between chaetocin and HSP90 in the context of treatment resistance. As such, our future research will focus on elucidating the role of chaetocin and HSP90 in mediating therapy resistance in lung cancer, which we believe could provide valuable insights into overcoming treatment challenges in this malignancy. Chaetocin disrupted the balance of cellular oxidative stress and metabolism through TKT to inhibit cell viability. Energy metabolic reprogramming is often seen as a novel marker of further complex changes in the tumor [35]. Metabolic reprogramming can promote tumor cell growth by producing more ATP, providing precursors for macromolecule synthesis, and reducing ROS production in tumor cells [36,37]. Our results indicated that chaetocin is able to stimulate ROS surge. Increased glycolysis is now often considered a hallmark of many cancers and is also used clinically as a diagnostic cancer biomarker and a target for cancer therapy. Metabolic reprogramming not only contributes to tumor resistance but also promotes cisplatin resistance by increasing glucose flux into PPP and NADPH production [38]. Our study showed that chaetocin can inhibit NADPH in a concentration-dependent manner. Many tumor cells show abnormally active PPP, especially nonoxidative branches, which may provide biosynthetic precursors such as ribose and nucleotides required by tumor cells to meet their rapidly growing demands [39]. We compared the expression of TKT in cisplatin-sensitive and cisplatin-resistant cells. It was found that TKT was highly expressed in cisplatin-resistant cells, which also indicated that the metabolism in drug-resistant cells was more active.

An essential enzyme in the PPP’s non-oxidative branch that connects the PPP to glycolysis is TKT. Sedoheptulose-7-phosphate and glyceraldehyde-3-phosphate are produced when TKT moves two carbon units from xylulose-5-phosphate (Xu5P) to ribose-5-phosphate. Fructose-6-phosphate (F6P) and glucose-3-phosphate are produced when TKT transfers two carbon atoms from Xu5P to erythrose-4-phosphate [40]. The enzymes representative for the oxidative or non-oxidative phase of PPP are glucose-6-phosphate dehydrogenase (G6PD) or TKT, respectively. The increased expression of G6PD can activate PPP, thereby reducing platinum-induced oxidative stress and gradually inducing tumor resistance [41]. Studies have shown that the PRMT-6PGD/ENO1 axis is critical for lung cancer progression, and inhibiting the signaling of this axis can enhance antitumor effect of cisplatin in preclinical lung cancer models [42]. Xanthatin, a sesquiterpene lactone isolated from Xanthatin leaves, can inhibit the expression of glucose transporter 1, thereby reducing PPP and leading to ROS accumulation and mitochondrial dysfunction in DDP-resistant lung cancer cells [43]. MiR-3663-5p can directly target TKT to diminish the nucleotide synthesis of the PPP pathway, thereby increasing gemcitabine sensitivity, which may be a supplement to chemotherapy advantages in pancreatic ductal adenocarcinoma [44]. Combining docetaxel and/or doxorubicin with a TKT inhibitor enhances the sensitivity of breast cancer cells to these two chemotherapeutic agents, thereby exerting a more potent inhibitory effect on cell proliferation [45]. The combination of imatinib (Gleevec, STI571) and the TKT inhibitor oxythiamine (OT) re-established the sensitivity of chronic myeloid leukemia cells to imatinib. Moreover, this combination led to a more pronounced suppression of tumor growth [46]. In general, TKT inhibition enhances the tumor-suppressive effect of cisplatin by modulating the levels of R5P, NADPH, and ROS, further promoting cisplatin-induced DNA damage. According to our existing experimental results, chaetocin can inhibit the enzyme activity and expression of TKT, and then inhibit NADPH, resulting in the surge of ROS and leading to apoptosis of drug-resistant tumor cells.

Finally, what is the connection between the target TKT and the signaling pathway? Through TKT knockdown experiments, we found that PI3K/Akt seemed to be regulated by TKT. Cheng et al. uncovered a reciprocal feedback mechanism between the PI3K/Akt pathway and PPP metabolism. This mechanism not only precisely regulates the distribution of glycolytic intermediates towards various branching metabolic pathways, thereby fulfilling the requirements for the rapid growth of tumor cells, but also strengthens the activation of the PI3K/Akt pathway and aerobic glycolysis, which is crucial for cancer metabolic reprogramming [47]. The PI3K/mTOR signaling pathway plays a key role in the development of primary and acquired radiation resistance in SCLC cells. PI3K/mTOR inhibitors make SCLC cells sensitive to radiation by promoting autophagy degradation of G6PD and aggravating oxidative stress damage [48]. TKT was significantly overexpressed in samples of oxaliplatin-resistant colorectal cancer patients [36,49]. TKT can activate the PI3K/Akt signaling pathway, thereby enhancing glucose uptake and lactate production in gastric cancer cells [22]. It has been reported that Akt and TKT are correlated, and TKT can promote the phosphorylation of Akt, which in turn enhances the metastatic ability of colon cancer [49,50]. This study provides insight into the link between the PI3K/Akt signaling pathway and potential chemotherapy resistance in PPP and provides mechanistic evidence supporting chaetocin as a possible therapeutic strategy to eliminate chemotherapy resistance in NSCLC.

## 5. Conclusions

Enhanced PPP metabolism enables DDP-resistant cells to better adapt to the oxidative stress induced via chemotherapy treatment. Our study showed that DDP-resistant NSCLC cells had upregulated TKT, and TKT was a promising target for DDP resistance in NSCLC. Chaetocin could directly bind to TKT to disrupt the balance of cellular oxidative stress and could inhibit cell proliferation by regulating the TKT/PI3K/Akt pathway.

## Data Availability

The data are contained within the article or Supplementary Materials.

## Supplementary Materials

The following supporting information can be downloaded at: https://www.mdpi.com/article/10.3390/antiox14030330/s1, Supplementary Figure S1: Chaetocin Induced Apoptosis in A549/DDP and H460/DDP and Inhibited NSCLC Cell Growth. (A–C) Cell viability of A549 (A), H460 (B), A549/DDP, and H460/DDP (C) cells was examined using a CCK-8 assay at various concentrations of DDP. (D) Cell viability of HBE and HaCaT cells was examined using a CCK-8 assay at various concentrations of chaetocin. (E) Cell viability of A549/DDP and H460/DDP cells was examined using a CCK-8 assay at various concentrations of chaetocin treatment within 24 h. (F) The tumor volume of mice. (G) Tumor inhibition rate was used to evaluate the inhibitory effect of chaetocin on tumor growth in vivo. TGI = (one-treatment group tumor weight/control group tumor weight) × 100%. (H) H and E staining results of vital organs (heart, lung, spleen, renal) in the A549/DDP xenograft mouse model; Supplementary Figure S2: Multi-omics cluster analysis. (A,B) Principal component analysis of groups treated with chaetocin and without chaetocin in transcriptome (A) and proteome (B); Supplementary Figure S3: 2D results of molecular docking, visualized with LigPlus.
