# Supplementary material for: Chaetocin, a Natural Inhibitor of Transketolase, Suppresses the Non-Oxidative Pentose Phosphate Pathway and Inhibits the Growth of Drug-Resistant Non-Small Cell Lung Cancer
Source: Antioxidants (Basel). 2025 Mar 11;14(3):330. doi: 10.3390/antiox14030330 (PMC11939327; doi:10.3390/antiox14030330)
Supplement: Supplementary file 1 [file antioxidants-14-00330-s001.zip › antioxidants-3421033-supplementary.pdf]

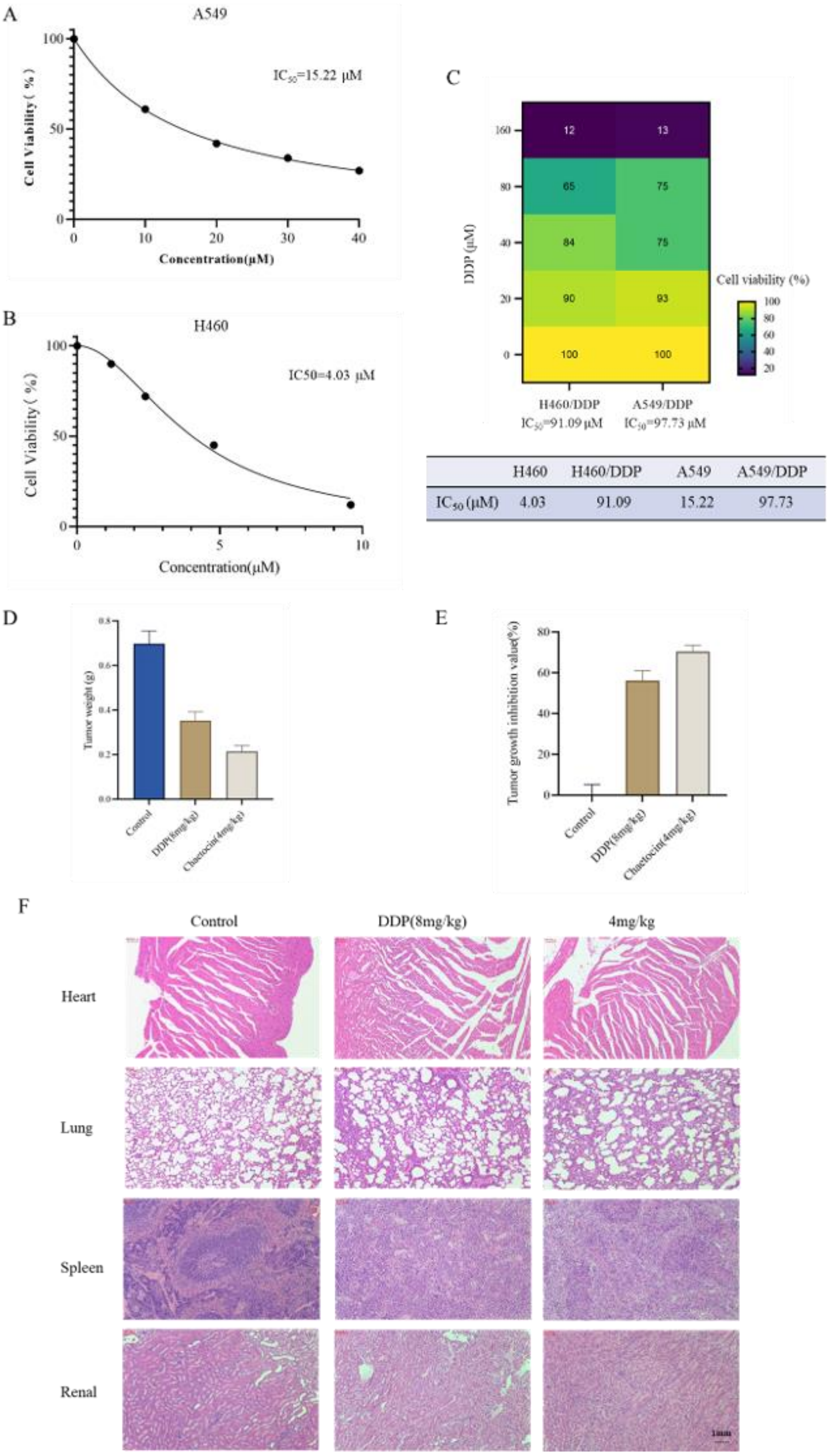

	H460	H460/DDP	A549	A549/DDP
IC_{50} (μM)	4.03	91.09	15.22	97.73

Supplementary Figure S1. (A-C) Cell proliferation of A549 (A), H460 (B), A549/DDP and H460/DDP (C) cells was examined using CCK-8 assay at various concentrations of DDP. (D) The tumor volume of mice. (E) Tumor inhibition rate was used to evaluate the inhibitory effect of chaetocin on tumor growth in vivo. TGI= (1-treatment group tumor weight/control group tumor weight) × 100%. (F) H&E staining results of vital organs (heart, lung, spleen, renal) in the A549/DDP xenograft mouse model.

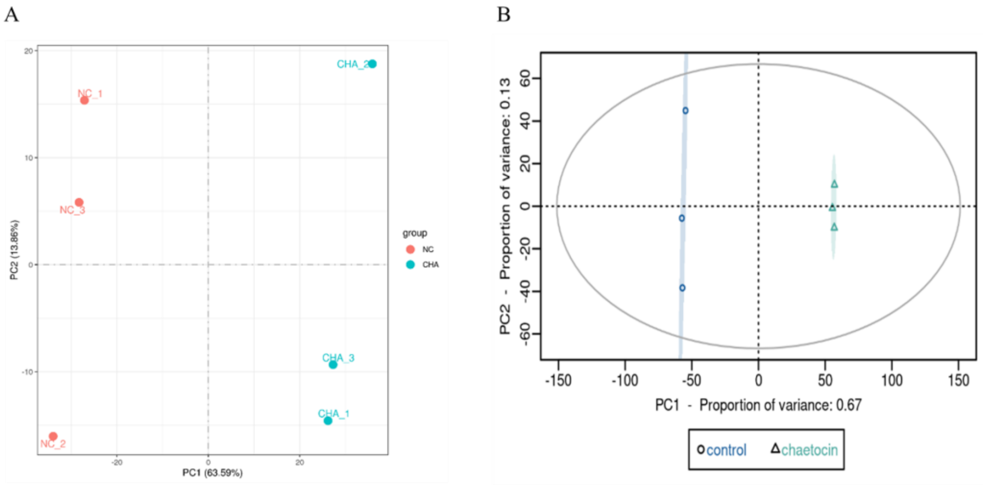

Supplementary Figure S2. (A, B) Principal component analysis of groups treated with chaetocin and without chaetocin in transcriptome (A) and proteome (B).

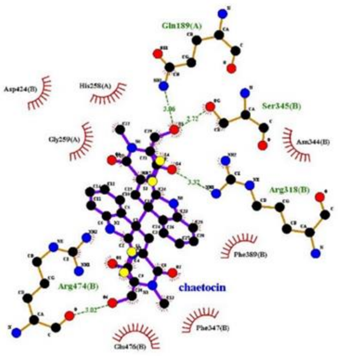

Supplementary Figure S3. 2D results of molecular docking, visualized with LigPlus.
